# Knock-down of YME1L1 induces mitochondrial dysfunction during early porcine embryonic development

**DOI:** 10.3389/fcell.2023.1147095

**Published:** 2023-04-13

**Authors:** Dongjie Zhou, Ming-Hong Sun, Wen-Jie Jiang, Xiao-Han Li, Song-Hee Lee, Geun Heo, Jungseok Choi, Kwan-Suk Kim, Xiang-Shun Cui

**Affiliations:** Department of Animal Science, Chungbuk National University, Cheongju, Chungbuk, Republic of Korea

**Keywords:** apoptosis, embryo development, fission, mitochondria, porcine, YME1L1

## Abstract

YME1L1, a mitochondrial metalloproteinase, is an Adenosine triphosphate (ATP)-dependent metalloproteinase and locates in the mitochondrial inner membrane. The protease domain of YME1L1 is oriented towards the mitochondrial intermembrane space, which modulates the mitochondrial GTPase optic atrophy type 1 (OPA1) processing. However, during embryonic development, there is no report yet about the role of YME1L1 on mitochondrial biogenesis and function in pigs. In the current study, the mRNA level of YME1L1 was knocked down by double strand RNA microinjection to the 1-cell stage embryos. The expression patterns of YME1L1 and its related proteins were performed by immunofluorescence and western blotting. To access the biological function of YME1L1, we first counted the preimplantation development rate, diameter, and total cell number of blastocyst on day-7. First, the localization of endogenous YME1L1 was found in the punctate structures of the mitochondria, and the expression level of YME1L1 is highly expressed from the 4-cell stage. Following significant knock-down of YME1L1, blastocyst rate and quality were decreased, and mitochondrial fragmentation was induced. YME1L1 knockdown induced excessive ROS production, lower mitochondrial membrane potential, and lower ATP levels. The OPA1 cleavage induced by YME1L1 knockdown was prevented by double knock-down of YME1L1 and OMA1. Moreover, cytochrome *c*, a pro-apoptotic signal, was released from the mitochondria after the knock-down of YME1L1. Taken together, these results indicate that YME1L1 is essential for regulating mitochondrial fission, function, and apoptosis during porcine embryo preimplantation development.

## 1 Introduction

Mitochondria are energy-supplying organelles that participate in diverse cellular processes and are important determinants for the oocyte maturation and embryo development in mammals (Dumollard et al., 2007; Van Blerkom and Davis, 2007). In oocytes, a greater number of mitochondria is essential for the high ability of oocyte maturation and embryo developmental (Reynier et al., 2001; Santos et al., 2006; Zeng et al., 2007). After fertilization, due to low mitochondrial biosynthetic activity, the number of mitochondria decreases and remains at a low level until the blastocyst stage (May-Panloup et al., 2005). In blastocysts, increased mitochondrial number can activate trophoblasts proliferation (Hendriks et al., 2019; Seli et al., 2019).

The mitochondrial metabolism is related to fusion and fission morphology transitions (Mishra and Chan, 2016). Mitochondrial fission is necessary to create new mitochondria, and autophagosomes can engulf dysfunctional mitochondria (Ashrafi and Schwarz, 2013). One of important regulators of mitochondrial dynamic on the inner membrane of mitochondria is the Dynamin-related GTPase optic atrophy type 1 (OPA1) (Delettre et al., 2000). There are two mitochondrial proteases including OMA1 (the ATP-independent protease) and YME1L (ATP-dependent AAA + protease). YME1L and OMA1 synergistically regulate mitochondrial morphology and balance fusion and fission through processing of OPA1 (the dynamin-like GTPase) (Anand et al., 2014). Moreover, YME1L promotes mitochondrial fusion, whereas OMA1 induces mitochondrial fragmentation by OPA1 processing (Quirós et al., 2012; Anand et al., 2014; Mishra et al., 2014). Both YME1L and OMA1 are degraded by different types of toxic insults (Rainbolt et al., 2016). The degradation of YME1L and OMA1 induced by different stress is a mechanism by which cells are sensitive to the proteolytic activity of the inner membrane of mitochondria and affects various aspects of mitochondrial biology in response to different types of stress. YME1L promotes the turnover of many proteins which are essential for the quality of mitochondria on both the inner membrane and intermembrane space of mitochondria (Rainbolt et al., 2013; Wai et al., 2016), and the lipid transfer proteins (Potting et al., 2013; Saita et al., 2018).

However, the effects of YME1L1, which regulates mitochondrial function and morphology during porcine embryo preimplantation development, have not been reported. In the present research, we hypothesized that YME1L1 is crucial for the porcine embryo preimplantation development by maintaining the mitochondrial morphology and the balance of fission and fusion. To evaluate this hypothesis, YME1L1 was knocked down using double-stranded RNA (dsRNA) at the zygote stage and cultured until the blastocyst stage for further analysis.

## 2 Materials and methods

### 2.1 Reagents

All reagents were purchased from Millipore Sigma (Burlington, MA, United States), and all manipulations of live embryos were performed on a heating plate at 38.5°C, unless otherwise indicated.

### 2.2 Porcine cumulus-oocyte complexes (COCs) collection and *in vitro* maturation (IVM)

Porcine ovaries were collected and stored at 37 °C in saline containing 75 mg/mL penicillin G and 50 mg/mL streptomycin sulfate and delivered from a local slaughterhouse (Farm Story Dodarm B&F, Umsung, Chungbuk, South Korea) to the laboratory. The diameter of follicles around 3–6 mm was aspirated using a 10 mL disposable syringe with an 18-gauge needle. The porcine COCs with more than two layers cumulus cells were collected and washed with HEPES briefly. Approximately 100 COCs were transported to each well of 4-well plates with IVM medium [TCM-199 (11150–059; Thermo Fisher Scientific, Waltham, MA, United States) containing 0.1 g/L sodium pyruvate, 10 ng/mL epidermal growth factor (EGF), 10 IU/mL follicle-stimulating hormone (FSH), 10 IU/mL luteinizing hormone (LH), and10% (v/v) porcine follicular fluid (PFF)] and incubated for 44–48 h at 38.5°C and 5% CO_2_.

### 2.3 Porcine MII stage oocytes parthenogenetic activation and *in vitro* culture (IVC)

The COCs with extended cumulus cells were pipetting for around 40–50 times in 1 mg/mL hyaluronidase to remove cumulus cells. MII stage oocytes with first polar body were then selected and parthenogenetically activated by 2 direct-current pulses of 120 V for 60 µs in activation buffer [297 mM mannitol (pH 7.2) supplemented with 0.1 mM CaCl_2_, 0.05 mM MgS0_4_, 0.01% polyvinyl alcohol (PVA, w/v), and 0.5 mM 4-(2-hydroxyethyl) piperazine-l-ethanesulfonic acid]. To prevent the pseudo-second polar body extrusion, the activated oocytes were treated with 7.5 μg/mL cytochalasin B for 3–6 h. Subsequently, approximately 50 embryos were washed and cultured in one of 4-well plates with IVC medium for microinjection. The embryos were then cultured for 7 days at 38.5°C and 5% CO_2_. The blastocysts on the 7^th^ day were randomly collected for further experiments. The total cell number of blastocyst was counted under the epifluorescence microscope (Nikon).

### 2.4 Preparation of YME1L1 double-stranded RNA (dsRNA)

The fragments of *YME1L1* and *OMA1*were amplified from cDNA of porcine COCs using the primers containing the T7 promoter sequence (Table 1). *In vitro* transcription was then performed using the MEGAscript T7 Kit (AM1333; Thermo Fisher Scientific) to synthesize dsRNA according to the manufacturer’s instructions. After 10 h *in vitro* transcription, dsRNA mixture was treated with DNase I for 15 min to degrade the DNA template, and then purified using Riboclear™ Plus (313–150; GeneAll Biotechnology). The purified dsRNA was dissolved in RNase-free water and stored at −80 °C until use.

**TABLE 1:** Summary of PCR primers.

Gene Name	Accession	Primer Sequence	Product Length	Used for
*YME1L1*	XM_021064882.1	F: TGT​TGG​TGG​GAA​GCG​AAT​TG	640	dsRNA
		R: CAT​GCT​GAC​GTC​CAT​CTG​TG		
*YME1L1*	XM_021064882.1	F: TTT​CTT​TTG​CGG​GAC​AGA​GG	631	dsRNA
		R: CAA​TTC​GCT​TCC​CAC​CAA​CAG		
*YME1L1*	XM_021064882.1	F: AAA​GCT​ACG​ATC​ATG​CCA​CG	114	RT-qPCR
		R: CAT​GCT​GAC​GTC​CAT​CTG​TG		
*OMA1*	XM_013999356.2	F: CAC​CCT​TCT​CAT​GGC​AAT​CG	730	dsRNA
		R: AAG​TCT​GTA​GCC​CAA​GGT​CC		
*18S*	NR_046261	F: CGC​GGT​TCT​ATT​TTG​TTG​GT	219	RT-qPCR
		R: AGT​CGG​CAT​CGT​TTA​TGG​TC		
*ND1*	NC_000845.1	F: CCT​ACT​GGC​CGT​AGC​ATT​CC	162	mtDNA copy number
		R: GAG​GAT​GTG​CCT​GGT​CGT​AG		

### 2.5 Microinjection

For the knock-down experiments, the dsRNA of *YME1L1* or *OMA1* was microinjected into 1-cell stage embryos after CB treatment using a Nikon Diaphot Eclipse TE300 inverted microscope (Nikon, Tokyo, Japan) connecting an Eppendorf Femto-Jet (Eppendorf, Hamburg, Germany) and equipped with a Narishige MM0-202N hydraulic 3-D micromanipulator (Narishige, Amityville, NY, United States). After microinjection, the embryos were continually cultured in IVC medium for 7 days. The dsRNA of green fluorescent protein (GFP) was microinjected as the control group.

### 2.6 Mitochondrial DNA copy number measurements

As previously reported (Niu et al., 2020), three blastocysts per tube were lysis at 65°C for 30 min and 95°C for 5 min in the buffer [20 mM Tris, 0.4 mg/mL proteinase K, 0.9% Nonidet-40, and 0.9% Tween 20]. After dilution 1:50 in ddH_2_O, real-time qPCR was performed using a pair of primers for *ND1* (Table 1).

### 2.7 ATP measurements

Ten blastocysts per tube were lysis with 30 µL buffer containing 20 mM Tris, 0.9% Nonidet-40, and 0.9% Tween 20 and then vortexed until no blastocyst is visible under the microscope. The ATP determination kit (A22066; Molecular Probes) were used for measurement, the standard reaction solution was prepared and placed on ice avoiding the light before use. Next, 5 µL lysate were transported to 96-well plates and equilibrated for 10 s, and 150 µL of the standard reaction solution was mixed with lysate in each well. ATP levels were measured using a lurninometer (CentroPro LB 962; Berthold Technologies, Bad Wildbad, Germany) for 10 s after 2 s delay. The signal intensity of YME1L1 knock-down group was relative to the control group which was arbitrarily set to one.

### 2.8 Measurement of mitochondrial ROS

Mitochondrial ROS were detected using MitoSOX Mitochondrial Superoxide Indicator (Thermo Fisher Scientific), as previously described (Nasr-Esfahani et al., 1990; Lee and Hyun, 2017). Briefly, ten blastocysts each group were treated with 10 µM MitoSOX indicator in the IVC medium for 30 min. Subsequently, the blastocysts were washed thrice with PBS/PVA and fixed in 3.7% paraformaldehyde for 30 min in the dark. TOM20 was stained according to the instruction in the immunofluorescence and confocal microscopy subsection. The fluorescence intensity of the mitochondria-derived ROS was analyzed using the ImageJ v. 44 g software (National Institutes of Health, Bethesda, MD, United States).

### 2.9 Mitochondrial membrane potential assay

The 7^th^ day blastocysts were treated with 2.5 µM 5,5′,6,6′-tetrachloro-1,1′,3,3′-tetraemyl-imidacarbocyanine iodide (JC-1) (M34152; Thermo Fisher Scientific) in the IVC medium for 30 min at 38.5°C in 5% CO_2_ and visualized by the epifluorescence microscope (Nikon). The ratio of intensity of red fluorescence of the activated mitochondria (J-aggregates), to green fluorescence of less-activated mitochondria (J-monomers) was calculated as the membrane potential (Sutton-McDowall et al., 2012) using ImageJ v. 44 g software (National Institutes of Health, Bethesda, MD, United States).

### 2.10 TUNEL assay

Following the instructions of the *In Situ* Cell Death Detection Kit (11684795910; Roche, Basel, Switzerland) (Ferris et al., 2016), ten blastocysts in each group were fixed in 3.7% paraformaldehyde for 30 min at room temperature. Subsequently, the blastocysts were washed trice with PBS/PVA and permeabilized using 1% Triton X-100 for 30 min at room temperature. Next, the blastocysts were fluorescein-conjugated dUTP and terminal deoxynucleotidyl transferase for 2 h. After washing trice with PBS/PVA, the blastocysts were mounted onto slides with VECTASHIELD^®^ Antifade Mounting Medium containing DAPI (H-1200; Vector Laboratories) and visualized by using a confocal microscope (LSM 710 Meta; Zeiss). The number of TUNEL-positive blastomeres was counted under the epifluorescence microscope (Nikon). The ratio of TUNEL-positive to the total blastomeres was indicated as apoptosis index.

### 2.11 EdU assay

The BeyoClick™ EdU Cell Proliferation Kit with Alexa Fluor 647 (Beyotime) was used for the cell proliferation assay. Briefly, the morula stage embryos were incubated with 10 μM EdU for 10 h at 38.5 °C. Next, the blastocysts were fixed with 3.7% paraformaldehyde at room temperature for 30 min and permeabilized with 1% Triton X-100 for 30 min. After washing thrice with PBS/PVA, the blastocysts were incubated in 100 μL of click reaction cocktail and 5 μg/mL of Hoechst 33342 in the dark for 30 min. Images were captured after mounting in PBS/PVA using a confocal microscope (LSM 710 Meta; Zeiss). The number of EdU-positive blastomeres was counted under the epifluorescence microscope (Nikon). The ratio of EdU-positive blastomeres to the total number of blastomeres was calculated as the proliferation rate.

### 2.12 Mitochondria and cytochrome c colocalization assay

Ten blastocysts per group were treated with 500 nM MitoTracker Red CMXRos (M7512; Thermo Fisher Scientific) in IVC medium at 38.5°C for 30 min. After washing thrice with PBS/PVA, blastocysts were fixed, and cytochrome *c* was stained following the instruction in the immunofluorescence and confocal microscopy subsection.

### 2.13 Immunofluorescence and confocal microscopy

Approximately ten embryos in each group were fixed in 3.7% paraformaldehyde at room temperature for 30 min, permeabilized with 1% Triton X-100 in the PBS/PVA for 30 min, and then blocked in 3% BSA containing 0.1% Triton X-100 in the PBS/PVA for 1 h at room temperature. Subsequently, the embryos were incubated with 1^st^ antibody at 4 °C overnight including anti-YME1L1 (1:50, 11510-1-AP; Proteintech, Cambridge, UK), anti- TOM20 (1:50, F-10, SC-17764; Santa Cruz Biotechnology, Dallas, TX, United States), anti-cytochrome *c* (1:50; ab110325; Abcam) and anti-cleaved caspase-3 (1:50; 9664S; Cell Signaling) diluted in blocking solution (3% BSA and 0.1% Triton X-100). After washing more than thrice with PBS/PVA, the embryos were incubated with 2^nd^ antibody such as Donkey anti-Mouse IgG, Alexa Fluor™ 488 (1:200; A-21202; Thermo Fisher Scientific), Goat anti-Rabbit IgG, Alexa Fluor™ 488 (1:200; A-11034; Thermo Fisher Scientific) or Donkey anti-Rabbit IgG, Alexa Fluor™ 546 (1:200; A10040; Thermo Fisher Scientific) for 1 h at room temperature. After washing thrice with PBS/PVA, the embryos were then mounted onto slides using VECTASHIELD^®^ Antifade Mounting Medium with DAPI (H-1200; Vector Laboratories) and captured using the confocal microscope (Zeiss LSM 710 Meta). Images were processed using the Zen software (v.8.0; Zeiss).

### 2.14 Real-time RT-PCR

According to the manufacturer’s instructions, approximately 30 embryos in each group were collected for mRNA extraction using the DynaBeads mRNA Direct Kit (61012; Thermo Fisher Scientific). Reverse transcription was performed using the LeGene Express 1st Strand cDNA Synthesis System (6210–05; LeGene Bioscience). Real-time RT-PCR for the *YME1L1* mRNA and *18S* rRNA (reference gene) was performed as follows: initial denaturation at 95°C for 3 min, followed by 40 repeats of cycle for the denaturation at 95°C for 15 s, annealing at 60°C for 25 s, and extension at 72°C for 15 s, and a final extension at 72°C for 5 min. The data were quantified using the 2^−ΔΔCT^ method (Livak and Schmittgen, 2001).

### 2.15 Western blot analysis

Approximately one hundred embryos in each group were lysis with 10 μL RIPA buffer and 10 μL 2X loading buffer on ice. After boiling the samples for 10–15 min, protein samples were separated by the different concentration of SDS-PAGE gel depends on the size of target protein and transferred onto polyvinylidene fluoride membranes for 1 h under 250 mA. Subsequently, the membranes were blocked with 5% skim milk in TBST buffer at room temperature for 1 h and then incubated with first antibody at 4°C overnight such as anti - YME1L1 (1:1000, 11510-1-AP; Proteintech), anti - DRP1 (1:1000; ab184247; Abcam), anti - LC3B (1:1000; NB100-2220; Novus Biological, Littleton, CO, United States), anti - OPA1 (1:1000, 27733-1-AP; Proteintech), anti - OMA1 (1:1000, sc-515788; Santa Cruz Biotechnology), anti-cleaved caspase-3 (1:1000; 9664S; Cell Signaling) or anti - GAPDH (1:1000; 5174S; Cell Signaling). After washing with TBST buffer for three times, the membranes were treated with secondary antibody for 1 h at room temperature such as horseradish peroxidase-conjugated goat anti-mouse or goat anti-rabbit IgG (1:20000; Santa Cruz Biotechnology). The membranes were then exposed using a UviSoft software (Uvitec, Cambridge, UK) with SuperSignal™ West Femto Maximum Sensitivity Substrate (Thermo Fisher Scientific, Waltham, United States).

### 2.16 Cytosol and mitochondrial fraction isolation

According to the manufacturer’s protocol, cytosolic and mitochondrial proteins of 500 blastocyst stage embryos were extracted using the Mitochondria/Cytosol Fractionation Kit (Cat# ab65320, Abcam). The expression of glyceraldehyde 3-phosphate dehydrogenase (GADPH) was measured as the purity of mitochondria fraction by western blot analysis.

### 2.17 Statistical analysis

The statistical data analysis was carried out by one-way analysis of variance (ANOVA) or Student’s t-test. All data were presented as the mean ± SEM. *p* < 0.05 was considered statistical significance. SPSS software v.19 (IBM SPSS, Chicago, IL, United States) was used for all calculations. All of experiments were replicated at least three times.

## 3 Results

### 3.1 Expression pattern of YME1L1 during early porcine embryonic development

**FIGURE 1:**
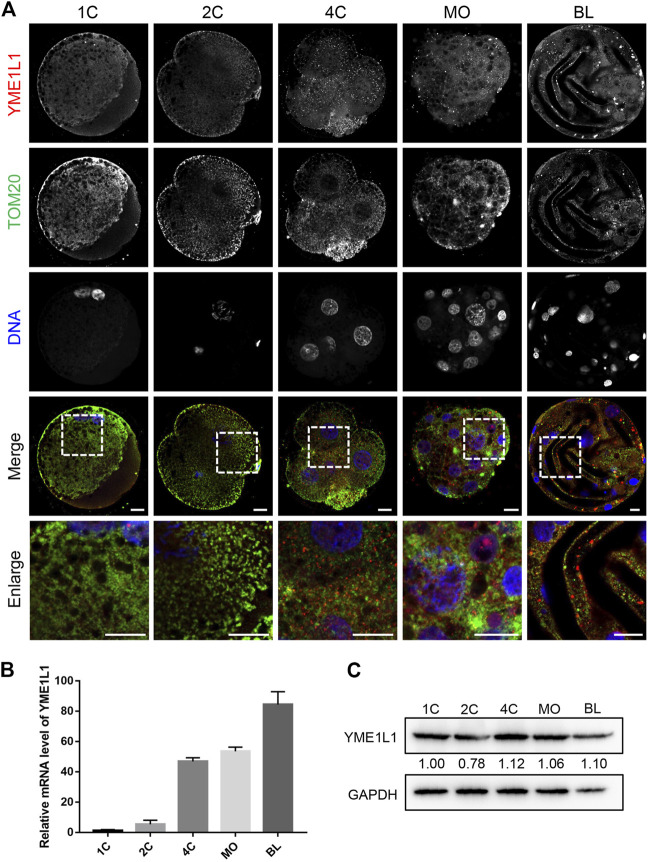
Expression of YME1L1 protein and mRNA during embryonic development in pig. **(A)** Representative confocal immunofluorescence images for YME1L1 and TOM20 expression at different stages during embryo development. **(B)** Quantitative PCR data of YME1L1 relative to the 1-cell expression level. **(C)** Western blot bands showed protein levels of YME1L1 in 1 at different stages during embryo development. Scale bar, 20 μm.

To evaluate the subcellular localization of YME1L1 during porcine embryo preimplantation development, YME1L1 and TOM20 were detected using immunofluorescence. The expression of YME1L1 was lower and colocalized with TOM20 in 1–2-cell stage embryos. However, YME1L1 was partially colocalized with TOM20 after the 4-cell stage at a higher level (Figure 1A). The mRNA expression of YME1L1 was detected by RT-qPCR and showed a gradual increase during embryo preimplantation development (Figure 1B). As shown in Figure 1C, the protein level of YME1L1 was verified by western blotting from 1-cell to blastocyst stage, which was highly expressed from 4-cell stage.

### 3.2 Effects of YME1L1 knock-down on early porcine embryonic development

**FIGURE 2:**
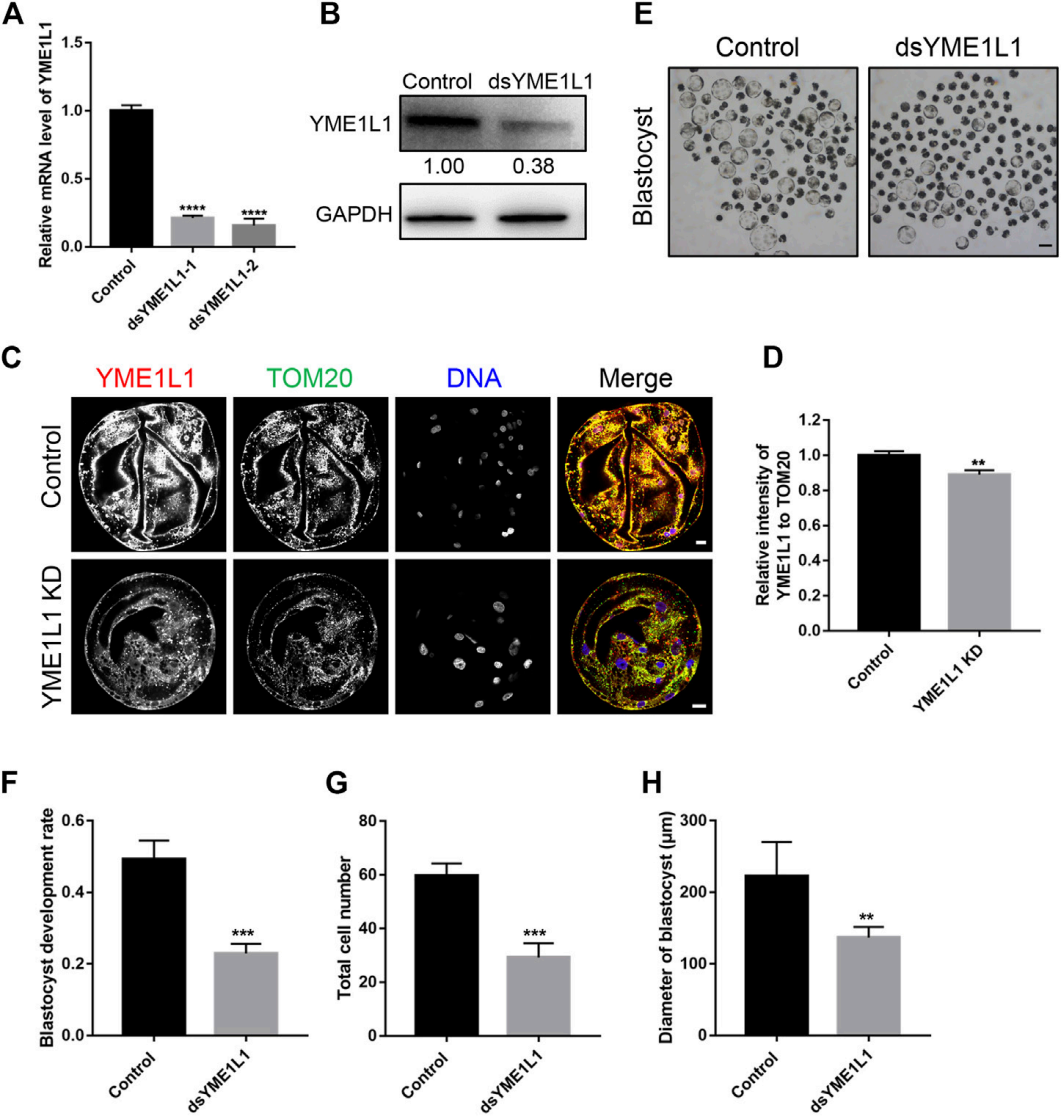
Effects of YME1L1 knock-down on embryonic development. **(A)** Quantitative PCR confirmed YME1L1 knock-down in the blastocyst stage embryos. **(B)** Protein levels of YME1L1 after YME1L1 knock-down at the blastocyst stage by western blot analysis. **(C, D)** Representative immunofluorescence images and relative intensity analysis of YME1L1 and TOM20 expression after YME1L1 knock-down at the blastocyst stage. **(E)** The morphology of the blastocyst stage embryos in both Control and YME1L1 KD groups. Scale bars, 100 μm. **(F)** The blastocyst rate in YME1L1 KD and Control groups. **(G)** Total cell number of YME1L1 KD (*n* = 25) and Control (*n* = 20) embryos. **(H)** Blastocyst diameter in the Control (*n* = 35) and YME1L1 KD (*n* = 20) group. **p < 0.01, ***p < 0.001 and ****p < 0.0001 were considered as significant differences.

To investigate the role of YME1L1 in porcine embryo preimplantation development, YME1L1 dsRNA (YME1L1 KD) was injected into 1-cell stage embryos, and then cultured in IVC medium for 7 days. The efficiency of *YME1L1* knock-down was analyzed at blastocyst stages (Figure 2A). Compared to the control group (GFP dsRNA), YME1L1 expression was significantly reduced after YME1L1 dsRNA microinjection (*p* < 0.001). YME1L1 knock-down was evaluated by western blotting and immunofluorescence, which showed that the protein level of YME1L1 was also significantly reduced in YME1L1 KD blastocysts (Figures 2B–D, *p* < 0.05). The ability of development to blastocyst stage was significantly decreased after YME1L1 knock-down (control: 46.33 ± 5.23 vs. YME1L1 KD: 21.67 ± 3.63, *p* < 0.005) (Figures 2E, F). Additionally, blastocyst quality in the YME1L1 KD group was worse than that in the control group (Figures 2G, H). YME1L1 KD significantly induced smaller diameter and less total cell number of blastocysts compared with control group (142.33 ± 8.92 vs. 217.67 ± 24.71 μm, *p* < 0.01; 30.33 ± 5.23 vs. 59.33 ± 3.67, *p* < 0.005). These data suggest that defective YME1L1 may disrupt early embryo development.

### 3.3 Knock-down of YME1L1 induces mitochondrial fragmentation

**FIGURE 3:**
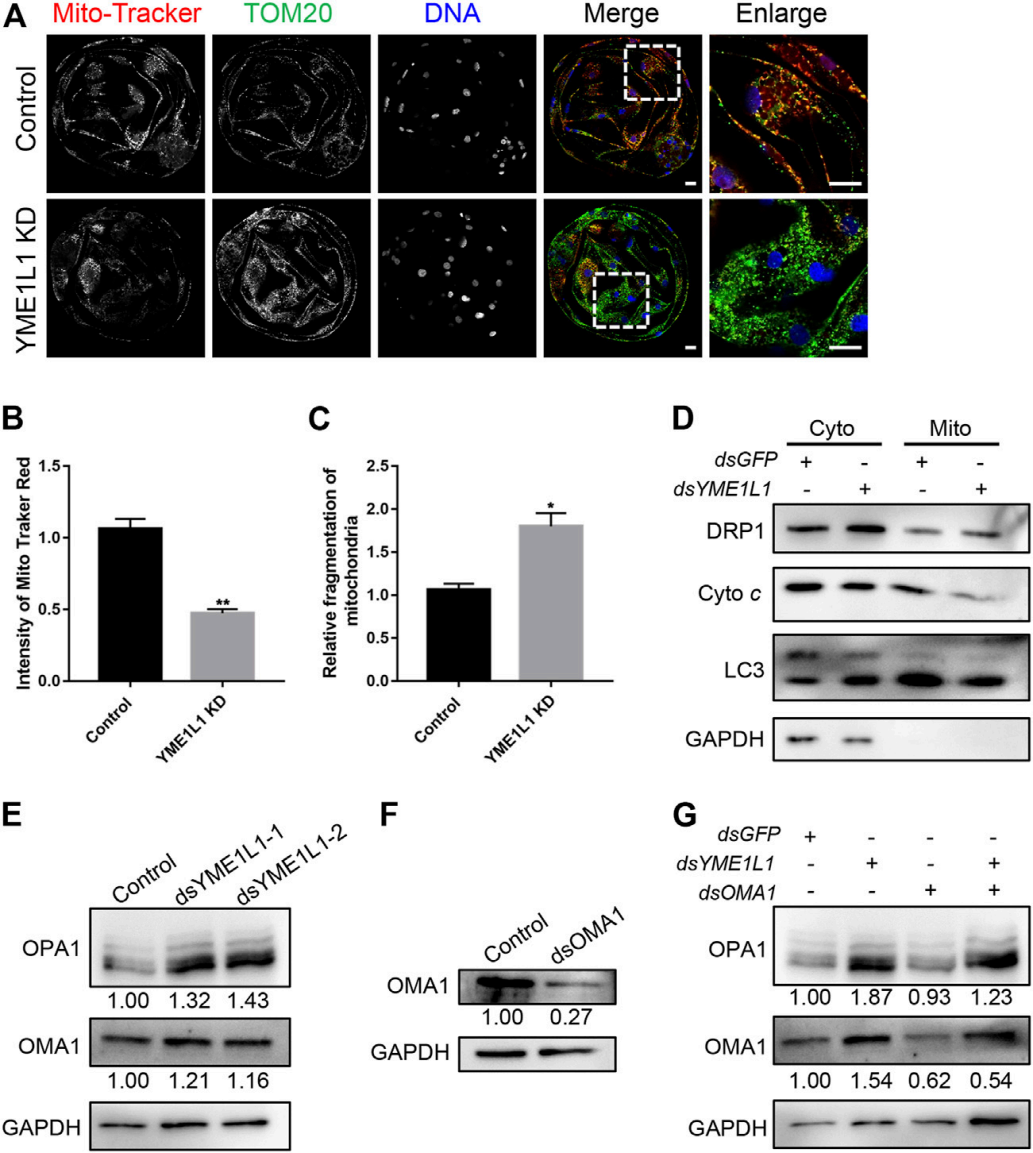
Knock-down of YME1L1 induces mitochondrial fragmentation and mitochondrial fission in embryos. **(A)** Representative immunofluorescence images for the mito-tracker and TOM20 in the Control and YME1L1 KD blastocysts. **(B, C)** Quantification of immunofluorescence images of Control (*n* = 10) and YME1L1 KD (*n* = 10) blastocyst stage embryos showing the mitochondria intensity and relative mitochondrial fragmentation rate, respectively. **(D)** Western blotting of blastocysts after isolation of cytosol and mitochondria fraction was performed to detect the level of DRP1, cytochrome *c* and LC3 in Control and YME1L1 KD group. **(E)** Protein levels of OPA1 and OMA1 after YME1L1 knock-down at the blastocyst stage by western blot analysis. **(F)** Protein levels of OMA1 after OMA1 knock-down at the blastocyst stage by western blot analysis. **(G)** Western blotting of the OPA1 and OMA1 level of blastocysts in Control, YME1L1 KD, OMA1 KD and Y + O KD (YME1L1 and OMA1 double knock-down) group. *p < 0.05 and **p < 0.01 were considered as significant differences. Scale bars, 20 μm.

To evaluate the effect of YME1L1 on the porcine embryo mitochondrial morphology after knock-down, the blastocysts were stained with TOM20 as total mitochondria and MitoTracker Red CMORos as active mitochondria. The intensity of MitoTracker and fragmentation of mitochondrial clusters were analyzed using ImageJ, the MitoTracker signal were highly distributed and colocalized with TOM20 in all control blastomeres. However, the active mitochondria were observed in partial blastomeres with lower intensity in YME1L1 KD group (*p* < 0.01, Figures 3A, B). Mitochondrial cluster fragmentation was significantly higher after knock-down of YME1L1 (*p* < 0.05, Figures 3A, C). Mitochondrial fragmentation is the result of increased fission in the mitochondria; thus, DRP1, a fission-related protein, was detected by western blotting. The bands showed that the level of DRP1 was higher when YME1L1 was knocked down in both cytosolic and mitochondrial fractions (Figure 3D). The balance of mitochondrial fusion and fission is important for mitochondrial function. The fragmented mitochondria induced by imbalance of mitochondrial fission and fusion leads to disease (Chan, 2006).

Additionally, YME1L1 KD induced more OMA1 due to the reduced degradation of OMA1 (Figure 3E). YME1L1 KD significantly increased the level of the short form of OPA1 (S-OPA1) compared with the control group (Figure 3E). Reduced OPA1 oligomerization can increase cellular sensitivity to apoptotic insults (Chung et al., 2006). To rescue this, dsRNA of OMA1 was co-injected into 1-cell stage embryos with dsRNA of YME1L1. The western blot results showed that OMA1 levels were significantly decreased and S-OPA1 levels were increased after dsRNA of OMA1 microinjection (Figures 3F, G). However, the expression of S-OPA1 and OMA1 was recovered by double knock-down of OMA1 and YME1L1 (Figure 3G).

### 3.4 Knock-down of YME1L1 induces dysfunction of mitochondria

**FIGURE 4:**
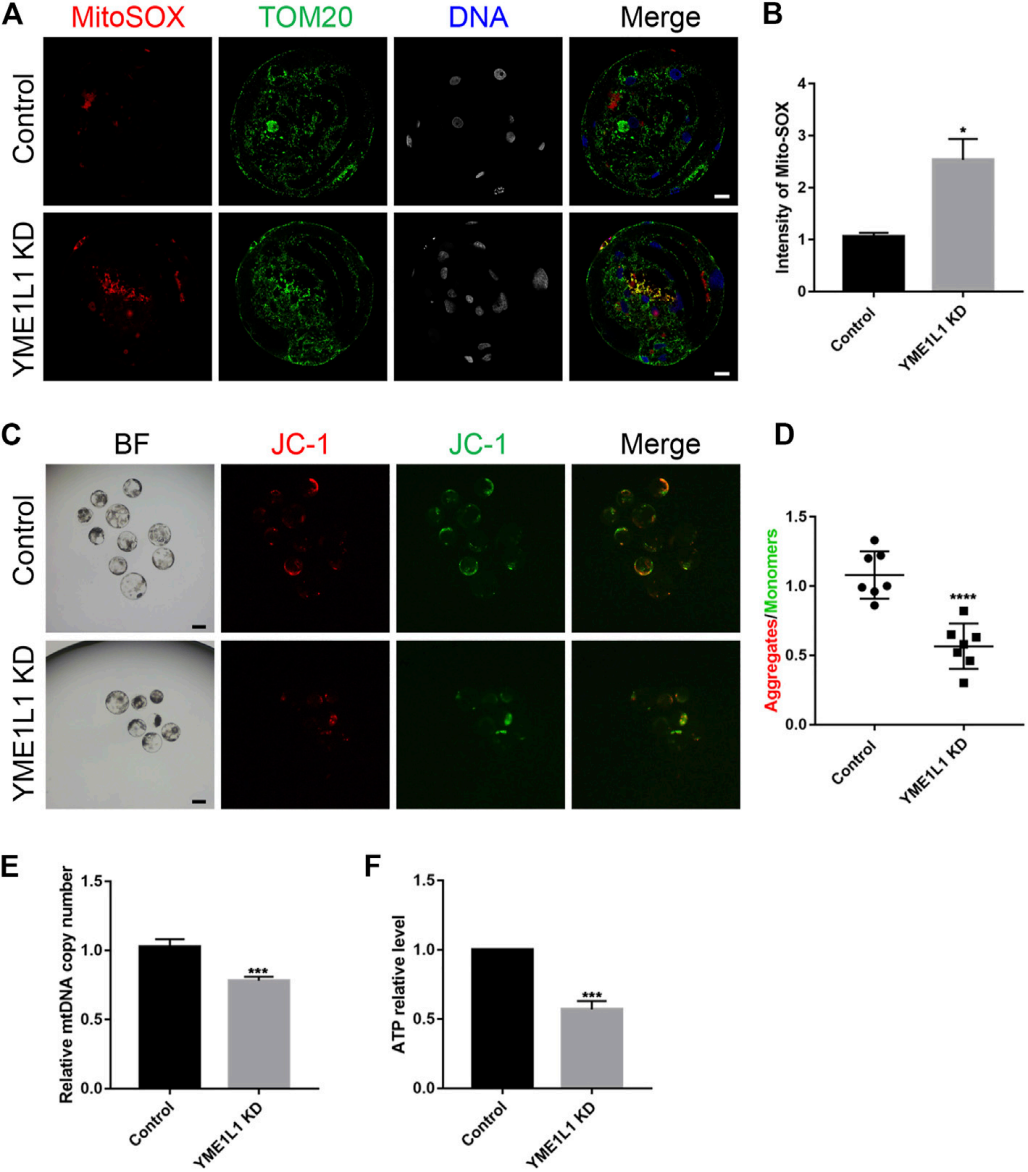
Knock-down of YME1L1 stimulates production of ROS and depolarizes mitochondrial membrane potential during embryo development. **(A, B)** Representative confocal images of Control and YME1L1 KD blastocysts showing MitoSOX and TOM20 staining and mitochondrial ROS levels. **(C, D)** JC-1 staining of blastocyst stage embryos in control and YME1L1 KD group. **(E)** Quantitative PCR confirmed relative copy numbers of mtDNA in control and YME1L1 KD group. **(F)** ATP levels at blastocyst stage in control and YME1L1 KD group. Scale bars, 20 μm *p < 0.05, ***p < 0.005, and ****p < 0.001 were considered as significant differences.

Based on the changes in the morphology of mitochondria, we next investigated if YME1L1 knock-down can promote the mitochondrial dysfunction in porcine embryos, which can be induced by ROS over generation, low mitochondrial membrane potential, and low ATP levels. Accordingly, mitochondrial ROS were detected at the blastocyst stage. As expected, compared to control blastocysts, mitochondrial ROS levels in YME1L1 KD blastocysts were significantly increased (*p* < 0.05, Figures 4A, B). Furthermore, quantification analysis of JC-1 staining showed that the ratio of green fluorescence to red fluorescence of the JC-1 dye was reduced by YME1L1 KD, compared with the control group (*p* < 0.001, Figures 4C, D), indicating that YME1L1 KD may induce mitochondrial membrane potential loss. Moreover, the relative mitochondrial DNA (mtDNA) copy number was quantified by RT-qPCR and showed more copy number of mtDNA in YME1L1 KD blastocysts than in the control blastocysts (Figure 4E). ATP levels were lower in YME1L1 KD blastocysts than in the control blastocysts (Figure 4F). These data indicated that knock-down of YME1L1 could induce mitochondrial fragmentation and ATP reduction.

### 3.5 Knock-down of YME1L1 induced apoptosis during porcine embryo preimplantation development

**FIGURE 5:**
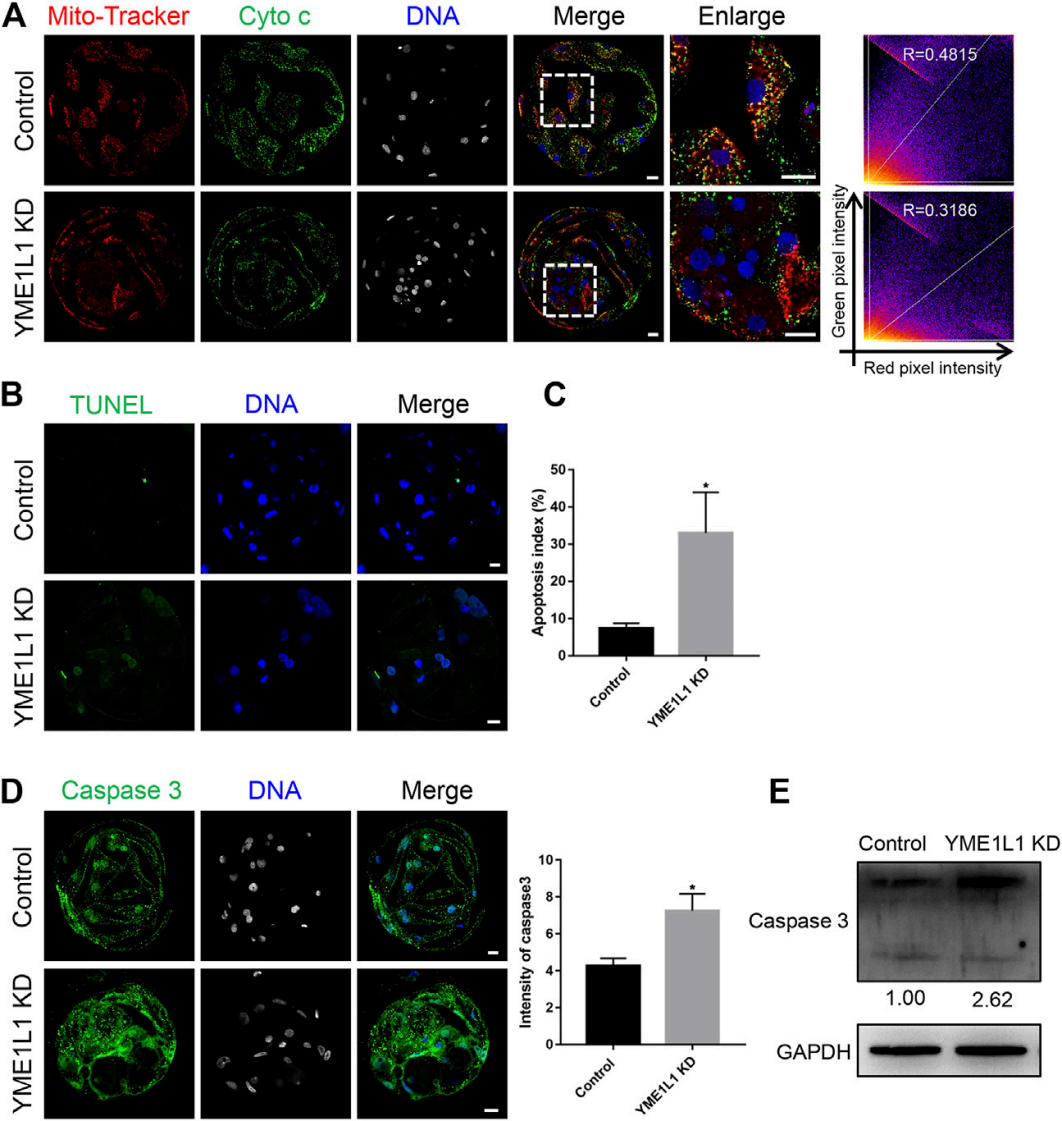
Knock-down of YME1L1 induces apoptosis in porcine embryos. **(A)** Representative images of blastocysts showed cytochrome *c* and MitoTracker Red staining in control and YME1L1 KD group. Scale bars, 20 μm. **(B, C)** TUNEL assay and apoptosis index at blastocyst stage in control and YME1L1 KD group. **(D)** Representative images of Control and YME1L1 KD blastocysts showed activated caspase 3 expression and relative intensity. Scale bars, 20 μm. **(E)** Western blotting of blastocysts in Control and YME1L1 KD group showing activated caspase 3 expression. *p < 0.05 indicate significant differences between treatment groups.

Previous research has demonstrated that cytochrome *c* is released from the mitochondria (Eleftheriadis et al., 2016). The colocalization of mitochondria and cytochrome *c* and the level of cytochrome *c* in mitochondria fraction at blastocysts stage was evaluated using immunofluorescence and western blotting after YME1L1 knock-down (Figure 3D; Figure 5A). Immunofluorescence images showed that co-localization of cytochrome *c* and MitoTracker was less in YME1L1 KD blastocysts, as detected by Mander’s overlap coefficient (Figure 5A). As shown in Figure 3D, fractionated mitochondrial and cytosolic proteins from control and YME1L1 KD blastocysts were analyzed for the content of cytochrome *c* by western blot. The cytochrome *c* in the cytosol remained relatively unchanged between the control and YME1L1 KD groups; however, mitochondrial cytochrome *c* decreased in the YME1L1 KD groups compared with the control.

As YME1L1 KD induced mitochondrial dysfunction and blastocyst total cell number reduction, the TUNEL assay was performed to evaluate whether apoptosis can be induced by the knock-down of YME1L1. The ratio of TUNEL-positive signal number to total nuclear number was significantly higher in YME1L1 KD blastocysts than in the control group (*p* < 0.05, Figures 5B, C). In addition, the active caspase 3 expression level was determined by immunofluorescence and western blotting, which showed a significant increase after the knock-down of YME1L1 (Figures 5D, E). Collectively, these results indicated that YME1L1 KD induces apoptosis during porcine embryo preimplantation development.

### 3.6 YME1L1 knock-down disrupts porcine embryo preimplantation development by inhibition of proliferation

**FIGURE 6:**
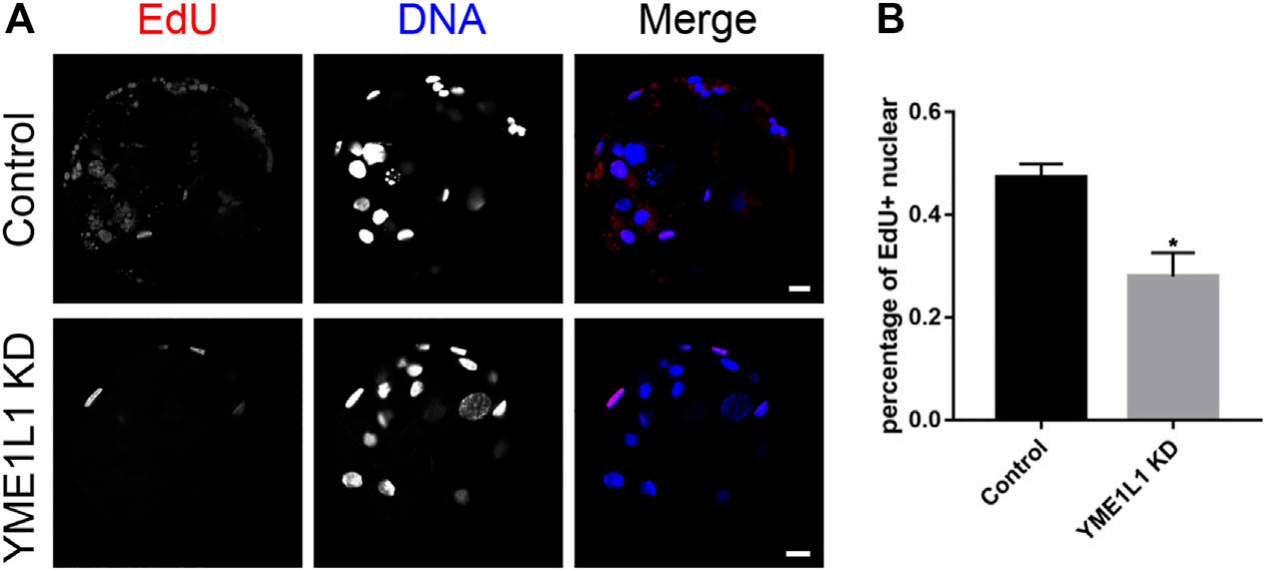
Knock-down of YME1L1 disrupts embryo development by inhibition of proliferation. **(A)** Representative images of Control and YME1L1 KD blastocysts showed EdU positive signals. Scale bars, 20 μm. **(B)** The ratio of EdU positive nuclear to total nuclear number (proliferative ability index) in Control and YME1L1 KD group. *p < 0.05 indicate significant differences between treatment groups.

Because of the poor developmental ability and poor quality of embryos after YME1L1 KD, we investigated whether YME1L1 could regulate embryo proliferation. As shown in Figures 6A, B, the percentage of EdU-positive nuclei was lower in the YME1L1 KD blastocysts than in the control group. This result suggested that YME1L1 KD-induced defects reduced the proliferation of porcine embryos.

## 4 Discussion

Previous work reported that mitochondria are important for the apoptosis, calcium homeostasis, cellular signaling pathways, gene expression, organic compound biosynthesis and thermogenesis (Gut and Verdin, 2013; Chandel, 2014; Shadel and Horvath, 2015). Approximately 1500 mitochondrial proteins are encoded by the nuclear genome and subsequently transported into the mitochondria. In the present study, we investigated the localization of YME1L1, which were co-localized with mitochondria before zygotic genome activation (ZGA, 4-cell stage in pigs). YME1L1 was also expressed in the cytoplasm after ZGA (Figure 1A), which demonstrates that newly synthesized YME1L1 is imported into the mitochondria. The protein level of YME1L1 was highly expressed from 4-cell stage (Figure 1C). These data indicate that high levels of YME1L1 after ZGA may be essential for embryo preimplantation development in pigs.

Low oocyte mtDNA content has been reported to result in abnormal embryo development in pigs (El Shourbagy et al., 2006). Therefore, a higher mtDNA copy number is essential for proper embryonic development (Wai et al., 2010). However, our results showed that few embryos in the YME1L1 KD group developed to the blastocyst stage (Figure 2F), and the mtDNA copy number was higher than that in control blastocysts (Figure 4E). This may be because the knock-down of YME1L1 disrupted the balance of mitochondrial fusion and fission. Because mitochondrial dynamics are regulated by GTPases, such as dynamin-related protein 1 (DRP1) and optic atrophy protein 1 (OPA1), we evaluated the levels of DRP1 and OPA1 by western blotting. Our data showed that both DRP1 and short form OPA1 were higher in the YME1L1 KD group than in the control (Figure 3D, E), which suggested that knockdown of YME1L1 can induce more mitochondrial fission than fusion. An insufficient ATP content has been considered one of the reasons for fertilization failure and abnormal embryo development (Van Blerkom et al., 1995). Therefore, mitochondrial mass must be sufficient to produce sufficient ATP and metabolites essential for embryonic development. Our results showed that knock-down of YME1L1 induced a reduction in ATP content in porcine embryos (Figure 4F). In addition, the LC3 level in both mitochondria and cytosol fraction was determined by western blotting, which showed higher expression after the knock-down of YME1L1 (Figure 3D). These data indicate that mitochondria induced by mitochondrial fission cannot produce enough ATP to support embryo development, because more mitophagy was induced by the knock-down of YME1L1.

The dynamic organization of mitochondria with a high membrane potential is crucial for embryo development (Au et al., 2005; Van Blerkom, 2009). Therefore, we evaluated the mitochondrial membrane potential after YME1L1 knock-down, and a lower in mitochondrial membrane potential was observed in the YME1L1 KD group (Figures 4C, D). In addition, ROS, as signals of mitochondrial dysfunction, plays a essential role in promoting apoptosis and cellular repair mechanisms (May-Panloup et al., 2021). When ROS are overproduced, different biomolecules are modified, and cellular damage is also induced (Cross et al., 1987; Guérin et al., 2001). Mitochondrial ROS levels were evaluated in blastocyst-stage embryos, which were increased by knock-down of YME1L1 (Figures 4A, B). Previous investigators have suggested that mitochondria is critical to the cell death pathways (Ong and Gustafsson, 2012; Vakifahmetoglu-Norberg et al., 2017). Mitochondrial fission and ROS over generation induce mitochondrial dysfunction in embryos (Frank et al., 2001; Youle and Karbowski, 2005; Yang et al., 2018), release of cytochrome *c* (Colombini, 2017). The release of cytochrome *c* promotes the caspase 3 activation, resulting in apoptosis (Seervi et al., 2011). In this regard, we evaluated apoptosis by measuring the cytochrome *c* release and the level of active caspase 3. We found that YME1L1 knock-down modulated the cytochrome *c* release (Figure 3D; Figure 5A) and induced higher level of the active caspase 3 in porcine embryos (Figures 5D, E). Moreover, during the embryo development, mitochondria play a role not only in the synthesis ATP, but also in maintaining redox homeostasis and producing intermediate metabolites (Guantes et al., 2016). We found that YME1L1 knock-down reduced embryo proliferation in the EdU assay (Figure 6).

## 5 Conclusion

**FIGURE 7:**
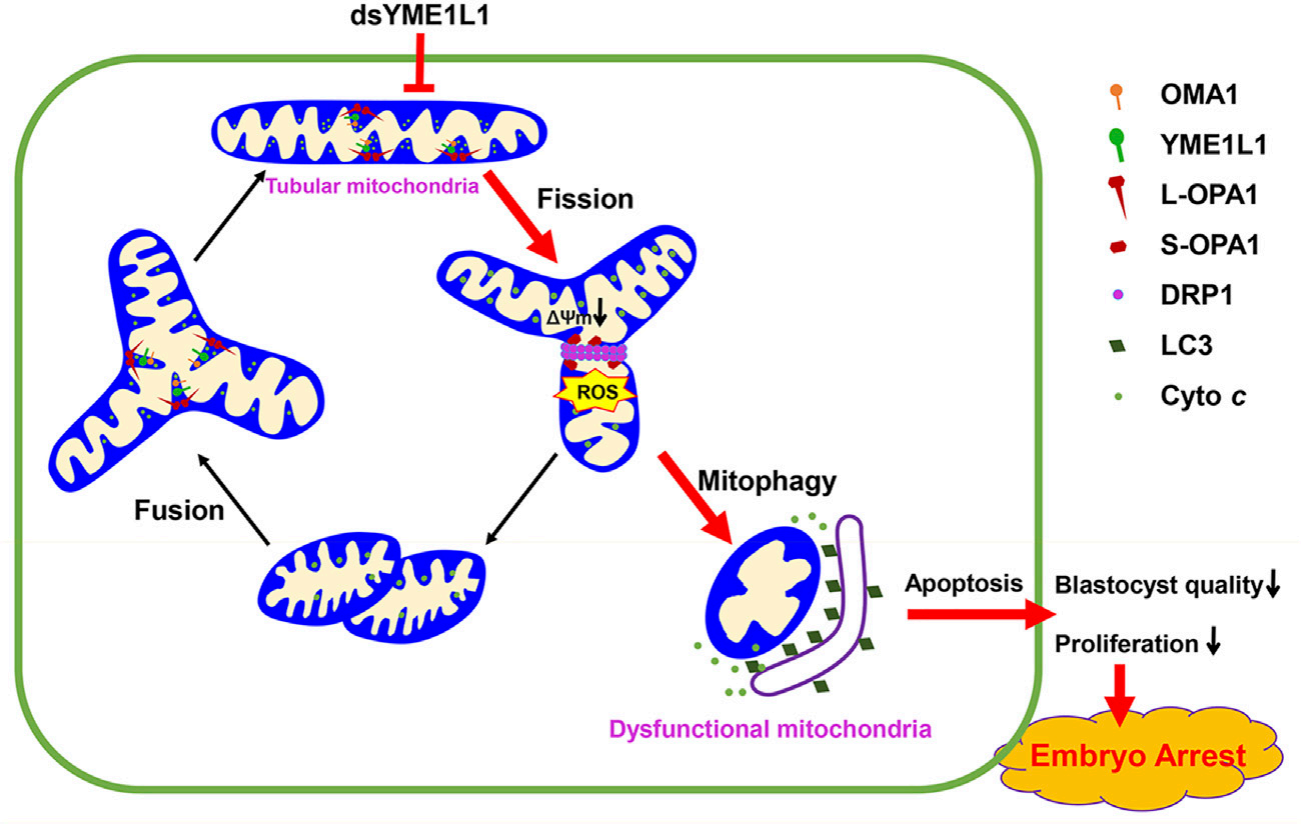
The balance of mitochondrial fusion and fission maintains functional morphology of mitochondria to support the porcine embryonic development. However, knock-down of YME1L1 during porcine embryo development results in mitochondrial dysfunction and fission which causes reduction of mitochondrial membrane potential and increased mitochondrial ROS generation, leading to mitochondrial fragmentation and apoptosis.

Overall, YME1L1 knock-down significantly reduced the blastocyst quality during porcine embryo preimplantation development (Figure 7). YME1L1 knock-down stimulates excessive ROS production, disrupts mitochondrial morphology and function, and increases cytochrome *c* release in porcine blastocysts. Moreover, the TUNEL and activated caspase-3 were induced by the knock-down of YME1L1 during porcine embryo preimplantation development. These results indicate that YME1L1 plays a crucial role in porcine embryo preimplantation development by modulating the oxidative stress, mitochondrial morphology and functions, and apoptosis.

## Data Availability

The original contributions presented in the study are included in the article/supplementary material further inquiries can be directed to the corresponding authors.
